# Supplementary material for: Emerging negative impact of warming on summer carbon uptake in northern ecosystems
Source: Nat Commun. 2018 Dec 19;9:5391. doi: 10.1038/s41467-018-07813-7 (PMC6300666; doi:10.1038/s41467-018-07813-7)
Supplement: Supplementary file 1 — Supplementary Information [file 41467_2018_7813_MOESM1_ESM.pdf]

Supporting Information for

Emerging negative warming impact on summer carbon uptake in northern ecosystems

Tao Wang[1,2*], Dan Liu[2], Shilong Piao[1,2,3], Yilong Wang[4], Xiaoyi Wang[2], Hui Guo[2], Xu Lian[3], John F Burkhart[5], Philippe Ciais[4], Mengtian Huang[3], Ivan Janssens[6], Yue Li[3], Yongwen Liu[3], Josep Peñuelas[7,8], Shushi Peng[3], Hui Yang[3], Yitong Yao[3], Yi Yin[4], Yutong Zhao[2]

[1] Center for Excellence in Tibetan Earth Science, Chinese Academy of Sciences, Beijing 100085, China

[2] Key Laboratory of Alpine Ecology, Institute of Tibetan Plateau Research, Chinese Academy of Sciences, Beijing 100085, China

[3] Sino-French Institute for Earth System Science, College of Urban and Environmental Sciences, Peking University, Beijing 100871, China

[4] Laboratoire des Sciences du Climat et de l'Environnement, CEA CNRS UVSQ, F-91191, Gif-sur-Yvette, France

[5] Department of Geosciences, University of Oslo, 0371 Oslo, Norway

[6] Department of Biology, University of Antwerp, Universiteitsplein 1, 2610 Wilrijk, Belgium

[7] CREAF, Cerdanyola del Valles, Barcelona 08193, Catalonia, Spain.

[8] CSIC, Global Ecology Unit CREAF-CEAB-CSIC-UAB, Cerdanyola del Valles, Barcelona 08193, Catalonia, Spain.

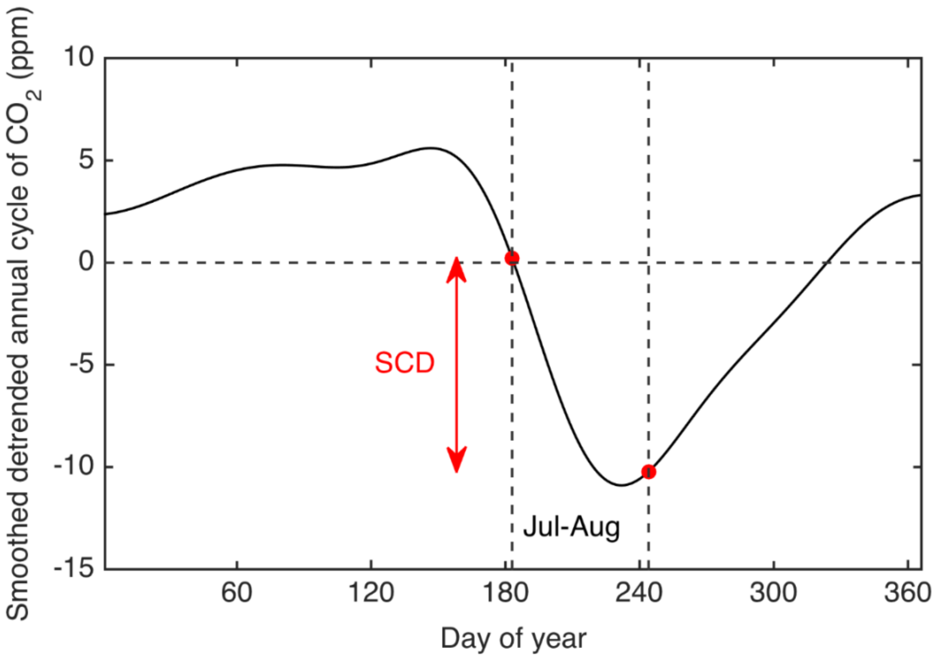

Supplementary Figure 1. Schematic of the definition of summer CO_2 drawdown. We used a smoothed time series of CO_2 concentrations for 2012 at Point Barrow (black line). The summer CO_2 drawdown (SCD), a proxy of net summer CO_2 uptake, was calculated as the difference between the CO_2 concentrations in the first week of July and the last week of August (red dots).

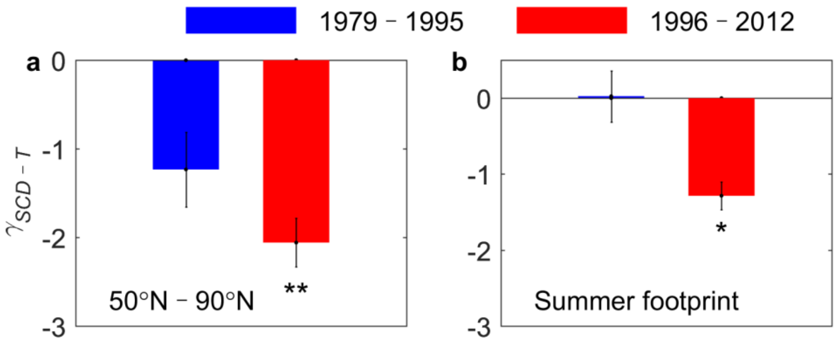

Supplementary Figure 2. The inter-annual sensitivity of summer CO_2 drawdown (SCD) to summer temperature (γ_{SCD-T}) for 1979–1995 (blue) and 1996–2012 (red) periods. The summer temperature is calculated as the average for July and August across ecosystems north of 50 °N (a), and the spatial average weighted by the potential emission sensitivities from FLEXPART over the vegetated land area within the multi-year mean summer footprint (b). We calculate $R_{SCD\text{-}T}$ through randomly selecting 14 of the 17 years in each corresponding period, and then take their standard deviation as the error bar. All variables were detrended for each period before the partial-correlation analysis. * and ** indicate that partial correlation coefficient is statistically significant at $P < 0.05$ and $P < 0.01$, respectively.

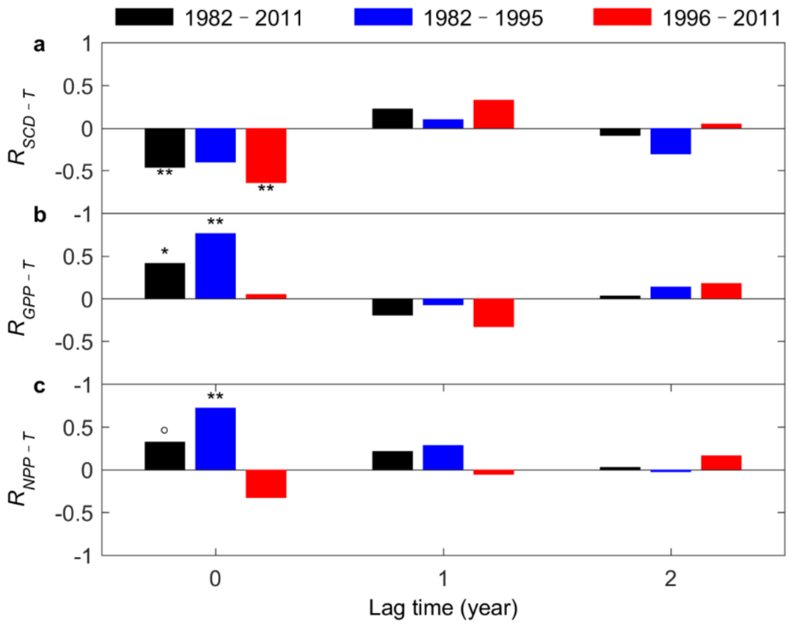

Supplementary Figure 3. The inter-annual partial correlation of summer CO_2 drawdown (SCD) (a), gross primary productivity (GPP) (b) and net primary productivity (NPP) (c) with different time lags (0-year, 1-year and 2-years) in summer temperature for 1982–2011 (black), 1979–1995 (blue) and 1996–2012 (red) periods. °, * and ** indicates that partial correlation coefficient is statistically significant at $P < 0.1$, $P < 0.05$ and $P < 0.01$, respectively.

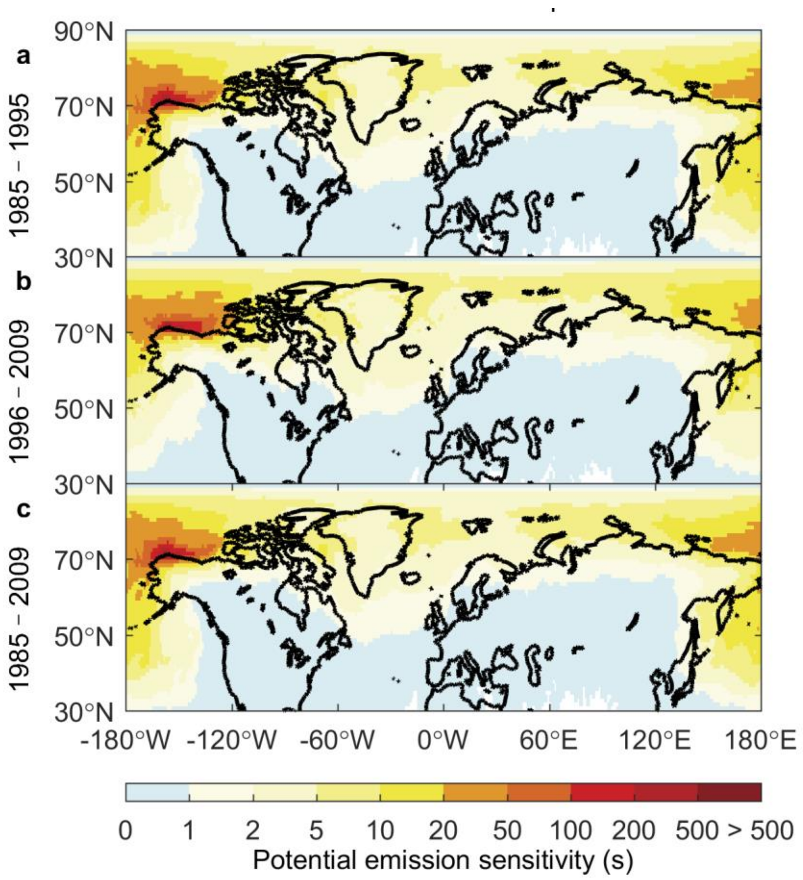

Supplementary Figure 4. Mean summer footprint area for Barrow derived from FLEXPART Lagrangian particle dispersion model during three time periods. The simulations were only available from 1985 to 2009.

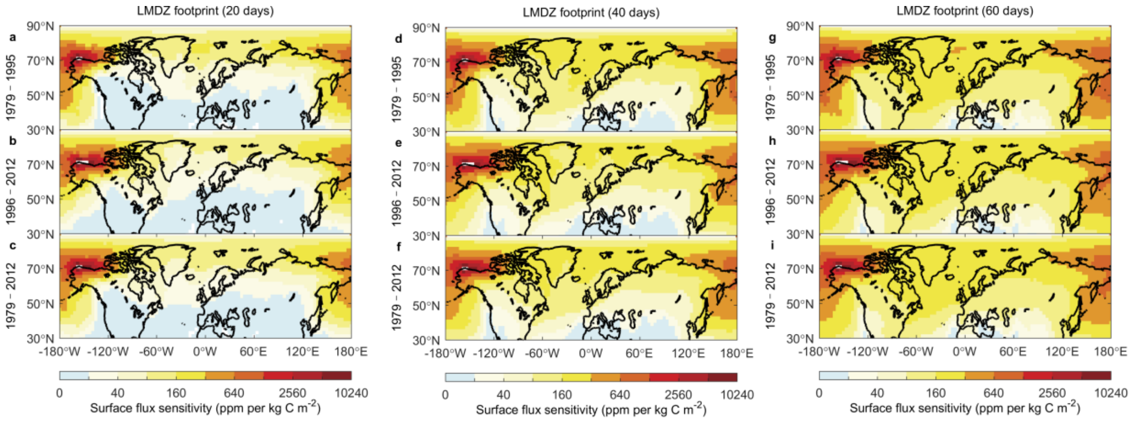

Supplementary Figure 5. Summer footprint area for Barrow derived from LMDZ model. The left (**a-c**), middle (**d-f**) and right (**g-i**) panel are footprint tracing 20, 40 and 60 days back from the last week of August, respectively.

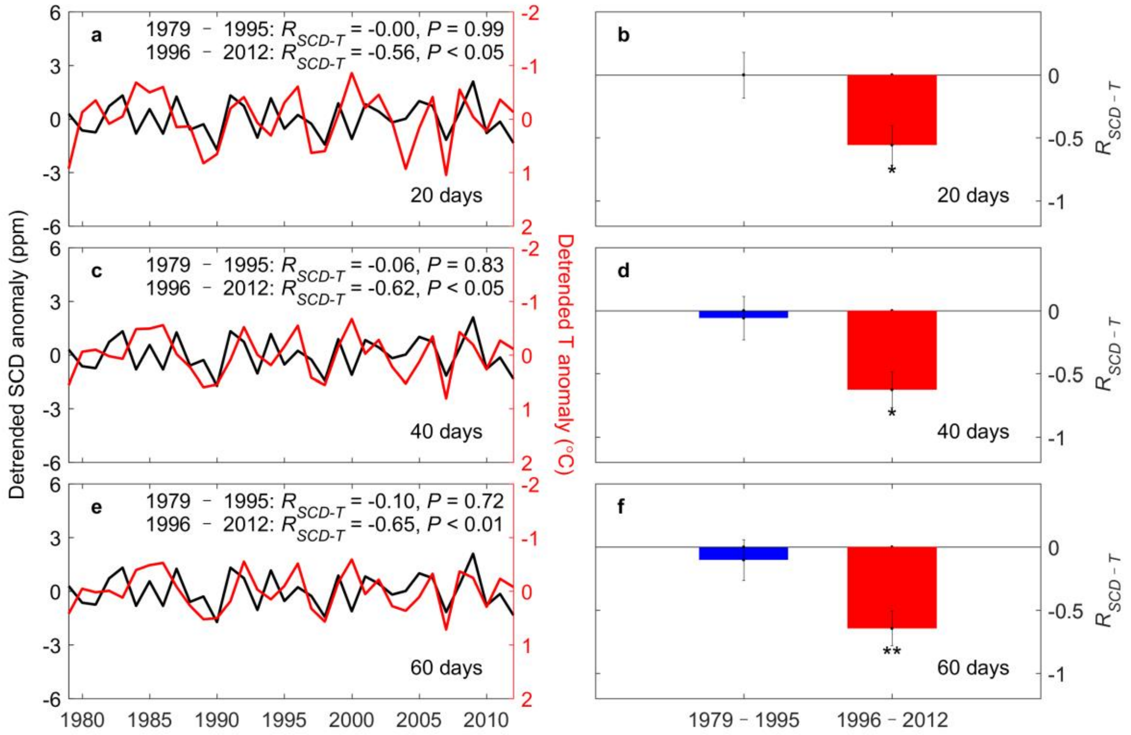

Supplementary Figure 6. The time series of detrended anomaly of summer CO_2 drawdown (SCD, black line) and summer temperature (T, red line) calculated as the average temperature for July and August over the main summer footprint region of Barrow CO_2 station. The summer footprint is calculated using LMDZ model with 20 days (a), 40 days (c) and 60 days (e) back-trajectory since the last week of August. The (**b**), (**d**), (**f**) illustrate the interannual partial-correlation coefficients between SCD and temperature (R_{SCD-T}), controlling for the effects of summer precipitation and cloudiness during 1979–1995 and 1996–2012. We calculate R_{SCD-T} through randomly selecting 14 of the 17 years in each corresponding period, and then take their standard deviation as the error bar. All variables were detrended for each period before the partial-correlation analysis. * and ** indicate that the partial-correlation coefficient is significant at $P < 0.05$ and $P < 0.01$, respectively.

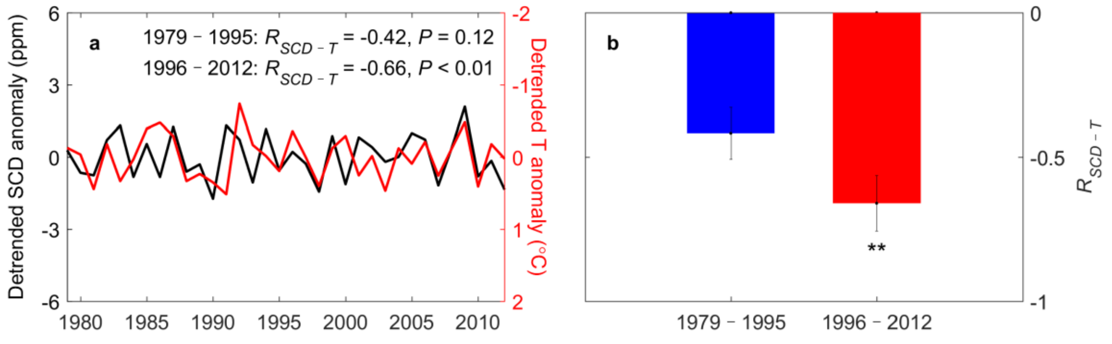

Supplementary Figure 7. Negative temperature control of summer CO_2 drawdown. Time series of anomalies of summer CO_2 drawdown (SCD, black line) and summer temperature (T, red line) calculated as the average for July and August across ecosystems north of 50 °N. Here we used summer temperature derived from the ERA-Interim data set. (**b**) illustrate the interannual partial-correlation coefficients between SCD and temperature ($R_{SCD\text{-}T}$), controlling for the effects of summer precipitation and cloudiness during 1979–1995 and 1996–2012. We calculate $R_{SCD\text{-}T}$ through randomly selecting 14 of the 17 years in each corresponding period, and then take their standard deviation as the error bar. All variables were detrended for each period before the partial-correlation analysis. ** indicate that the partial-correlation coefficient is significant at $P < 0.01$.

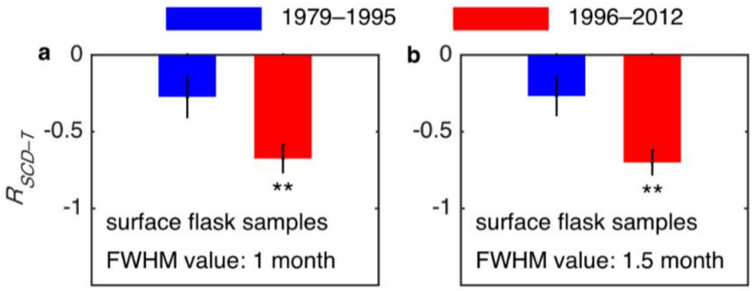

Supplementary Figure 8. The inter-annual partial correlation between summer CO_2 drawdown (SCD) and temperature ($R_{SCD\text{-}T}$) for 1979–1995 (blue) and 1996–2012 (red) periods. SCD was derived from surface-flask weekly samples using the 1-month FWHM averaging filter in (**a**) and from surface-flask weekly samples using the 1.5-month FWHM averaging filter in (**b**). We calculate $R_{SCD\text{-}T}$ through randomly selecting 14 of the 17 years in each corresponding period, and then take their standard deviation as the error bar. ** indicate that partial correlation coefficient is statistically significant at $P < 0.01$.

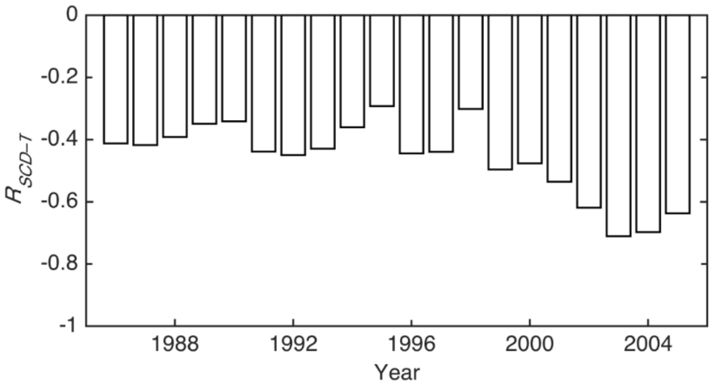

Supplementary Figure 9. Changes in the inter-annual partial correlation coefficient between summer CO_2 drawdown (SCD) and summer temperature (T, calculated as the average temperature for July and August from CRU) (R_{SCD-T}) after applying 15-year moving windows (all variables detrended for each corresponding window). The x axis is the central year of the 15-year moving window (for example, 2000 stands for a moving window from 1993 to 2007).

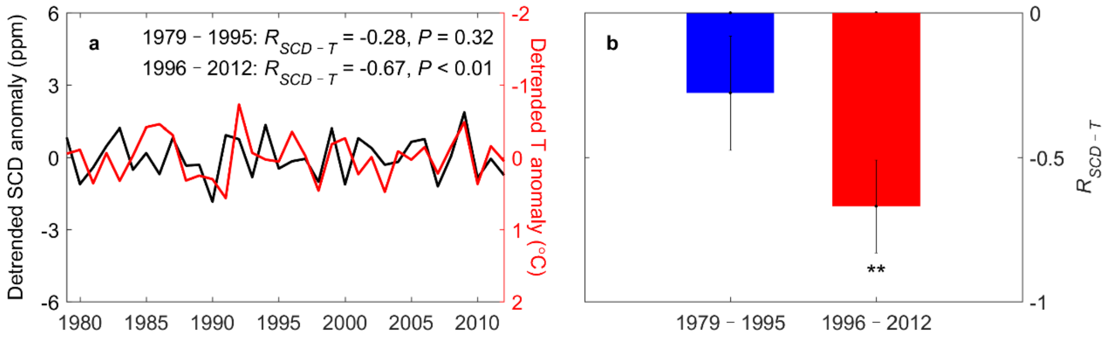

Supplementary Figure 10. Negative temperature control of summer CO_2 drawdown. (**a**) Time series of anomalies of summer CO_2 drawdown (SCD, black line) and summer temperature (T, red line). The summer CO_2 drawdown and summer temperature is calculated as the difference of CO_2 concentrations between the day of the year (DOY, 178) when detrended CO_2 crosses down its annual mean level and that (235) when detrended CO2 reaches its annual minimum. (**b**) illustrates the interannual partial-correlation coefficients between SCD and temperature (R_{SCD-T}), controlling for the effects of summer precipitation and cloudiness during 1979–1995 and 1996–2012. We calculate R_{SCD-T} through randomly selecting 14 of the 17 years in each corresponding period, and then take their standard deviation as the error bar. ** indicates that partial correlation coefficient is statistically significant at $P < 0.01$.

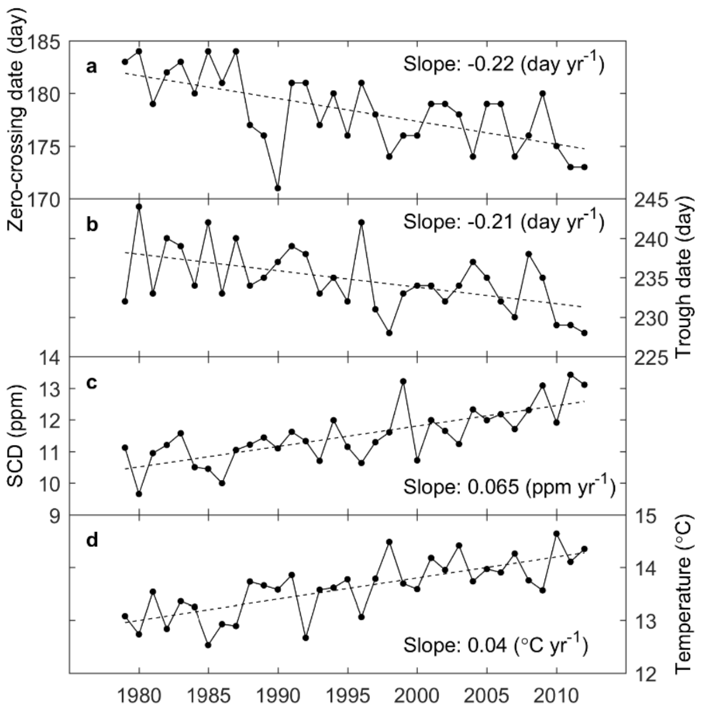

Supplementary Figure 11. The time series of spring zero-crossing date (a), the trough date (b), the SCD defined by inter-annually varying zero-crossing date and trough date (c) and the average temperature between inter-annually varying zero-crossing date and trough date (d).

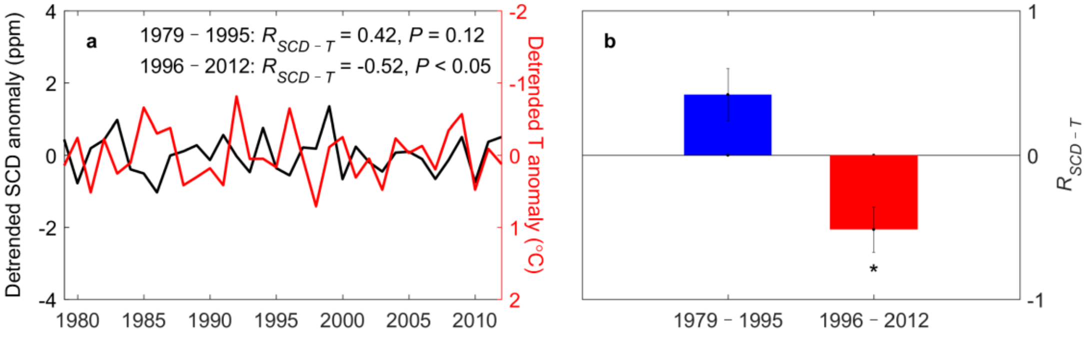

Supplementary Figure 12. Negative temperature control of summer CO_2 drawdown. (**a**) Time series of anomalies of summer CO_2 drawdown (SCD, black line) and summer temperature (T, red line). Here the summer CO_2 drawdown (SCD, black line) and summer temperature (T, red line) are calculated for the period between the inter-annual varying date when CO_2 crosses down zero (inter-annual varying spring zero-crossing date) and the inter-annual varying date when CO_2 reaches its annual minimum (inter-annual varying trough date). (**b**) illustrates the interannual partial-correlation coefficients between SCD and temperature ($R_{SCD\text{-}T}$), controlling for the effects of summer precipitation and cloudiness during 1979–1995 and 1996–2012. We calculate $R_{SCD\text{-}T}$ through randomly selecting 14 of the 17 years in each corresponding period, and then take their standard deviation as the error bar. All variables were detrended for each period before the partial-correlation analysis. * indicate that the partial-correlation coefficient is significant at $P < 0.05$.

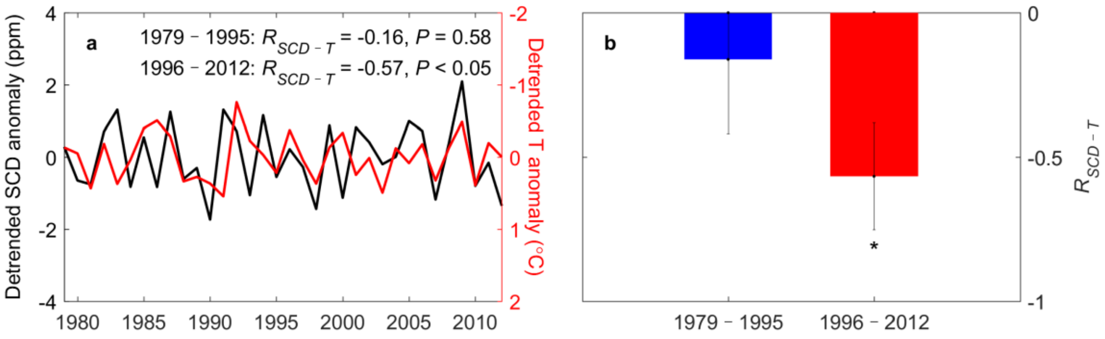

Supplementary Figure 13. Negative temperature control of summer CO_2 drawdown. Time series of anomalies of summer CO_2 drawdown (SCD, black line) and summer temperature (T, red line) calculated as the average for July and August across ecosystems north of 50 °N. Here we calculated partial correlation between SCD and air temperature with controlling precipitation, cloudiness and Arctic sea-ice extent (SIE) during summer (July and August). (**b**) illustrates the interannual partial correlation coefficient between SCD and SIE. We calculate R_{SCD-T} through randomly selecting 14 of the 17 years in each corresponding period, and then take their standard deviation as the error bar. * indicates that partial correlation coefficient is statistically significant at $P < 0.05$.

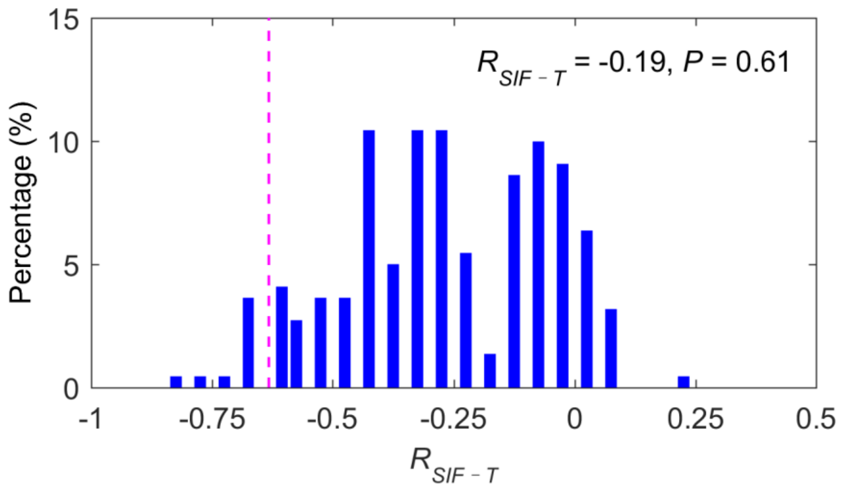

Supplementary Figure 14. The partial correlation between solar-induced chlorophyll fluorescence (SIF) and summer temperature for the period 2001–2012. Here we randomly selected 9 years from the time series for partial correlation analysis and the histogram shows the frequency distribution of the partial correlation coefficient between SIF and summer temperature. The magenta line illustrates the significance level at $P < 0.05$.

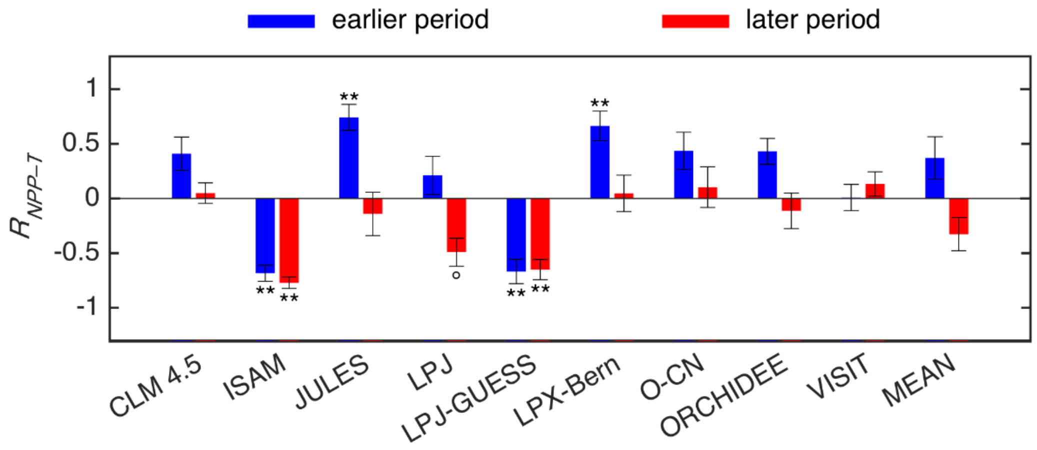

Supplementary Figure 15. Inter-annual partial correlation of net primary productivity (*$R_{NPP\text{-}T}$*) with summer temperature for 1979–1995 (blue) and 1996-2012 (red) periods for Trendy models. We calculate $R_{NPP\text{-}T}$ through randomly selecting 14 of the 17 years in each corresponding period, and then take their standard deviation as the error bar. °, * and ** indicates that partial correlation coefficient is statistically significant at $P < 0.1$, $P < 0.05$ and $P < 0.01$, respectively.

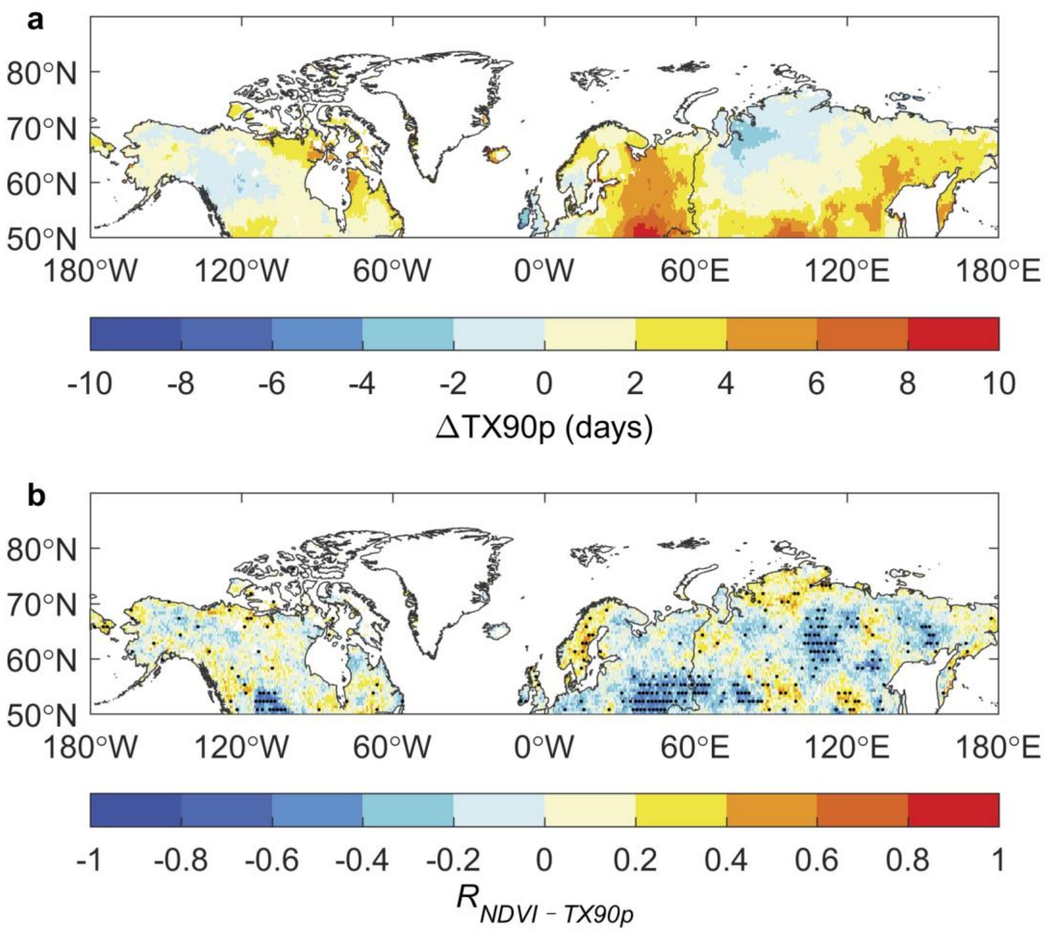

Supplementary Figure 16. Spatial distribution of the changes in extreme hot days during July and August (a) and the linkage between summer NDVI and extreme hot days (b). Extreme hot days (TX90p) were defined as the number of days when temperatures exceeded the 90th percentile, which was calculated based on the temperatures for 1982–2012. The changes are the difference between the 1996–2012 and 1982–1995 periods. The $R_{NDVI\text{-}TX90p}$ is defined as the partial correlation coefficient of NDVI against TX90p, whilst controlling for the effect of summer temperature. The gray dots in **b** indicate that the partial correlation coefficient is statistically significance ($P < 0.05$).

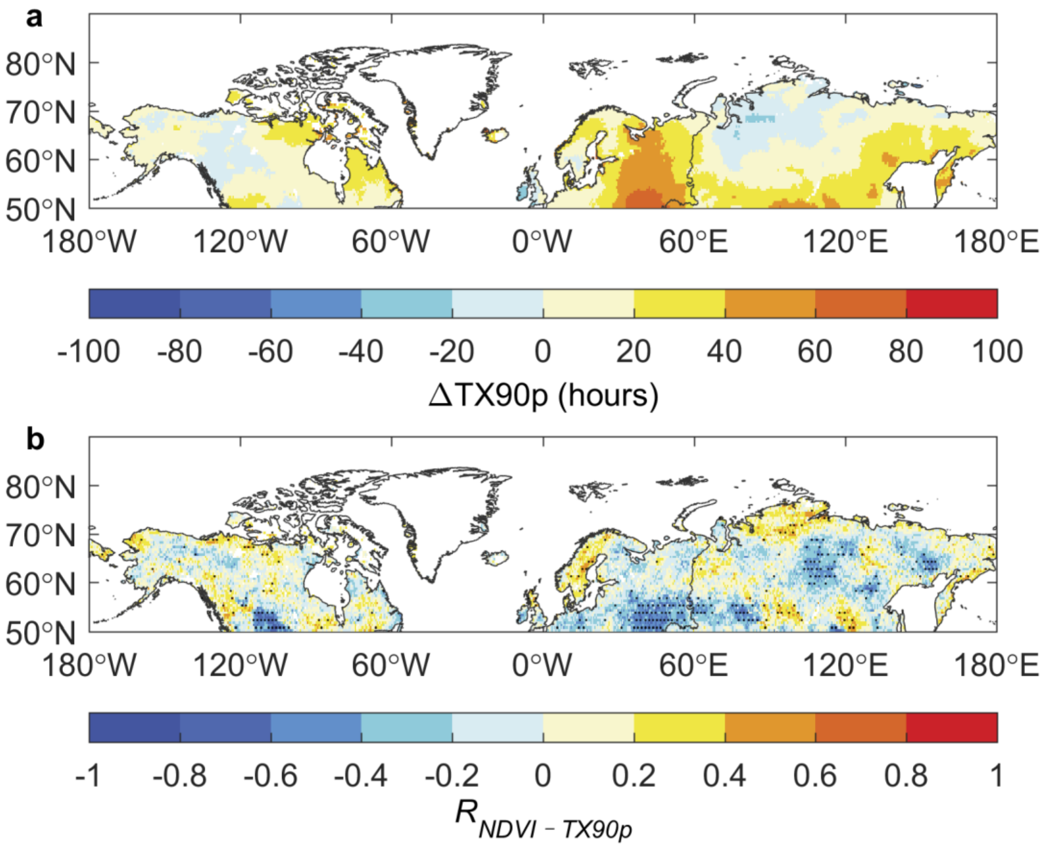

Supplementary Figure 17. Spatial distribution of the changes in extreme warm hours during July and August (a) and the linkage between summer NDVI and extreme warm hours (b). For each day of the summer season (July and August), we extracted the maximum temperature and its two neighbors based on 3-hourly temperature data from ERA-Interim data. The 3-hourly temperatures occurring throughout the entire study period 1979–2012 were sorted in ascending to determine the threshold value for the 90th percentile. The number of hours with temperatures exceeding 90th percentile were then referred to as extreme warm hours (TX90p). The changes are the difference between the 1996–2012 and 1979–1995 periods. The $R_{NDVI\text{-}TX90p}$ is defined as the partial correlation coefficient of NDVI against TX90p, whilst controlling for the effect of

summer temperature. The gray dots indicate that the partial correlation coefficient is statistically significance ($P < 0.05$).

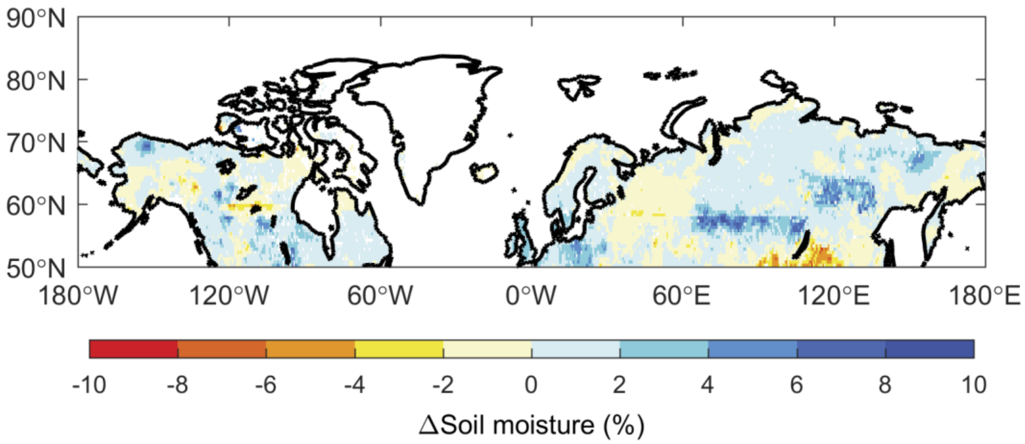

Supplementary Figure 18. Spatial distribution of the difference of root-zone soil moisture from the Global Land Evaporation Amsterdam Model (GLEAM) data set between the 1996–2012 and 1982–1995 periods.

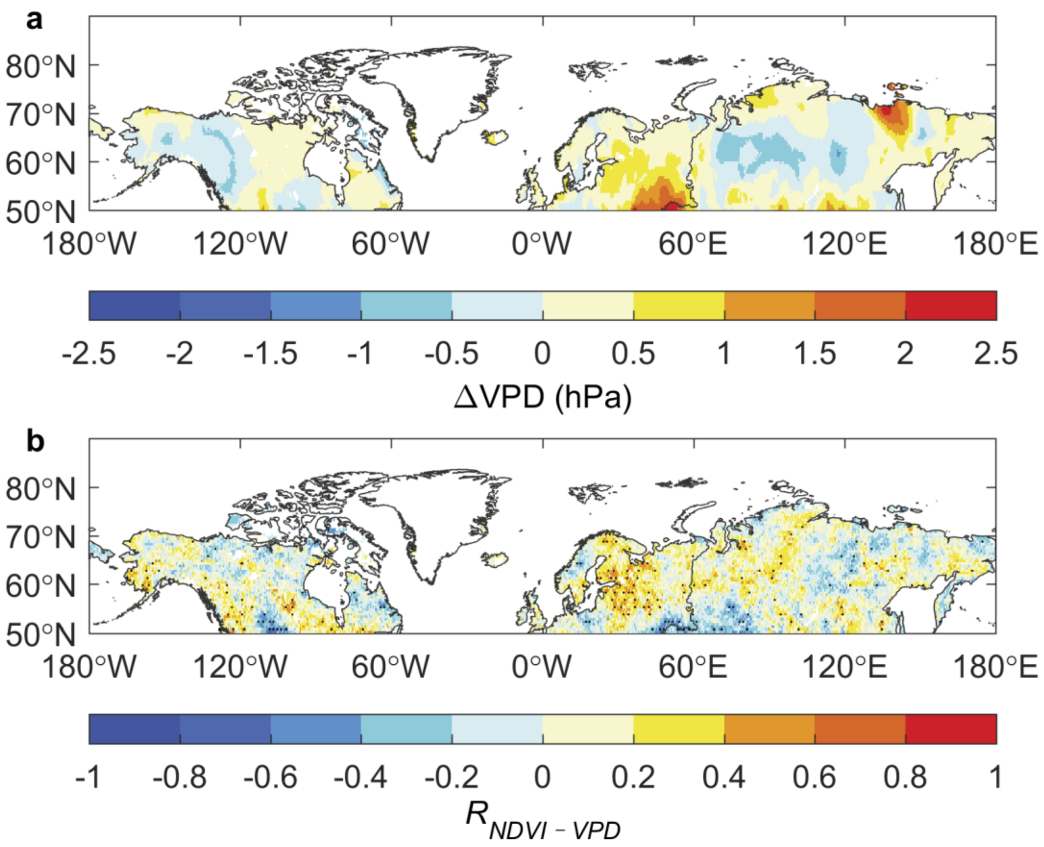

Supplementary Figure 19. Spatial distribution of changes in vapor pressure deficit (VPD) during July and August (a) and the linkage between summer NDVI and VPD (b). The VPD changes are the difference between the 1996–2012 and 1982–1995 periods. The $R_{NDVI\text{-}VPD}$ is defined as the partial correlation coefficient of NDVI against VPD, whilst controlling for the effect of summer temperature. The gray dots indicate that the partial correlation coefficient is statistically significance ($P < 0.05$).

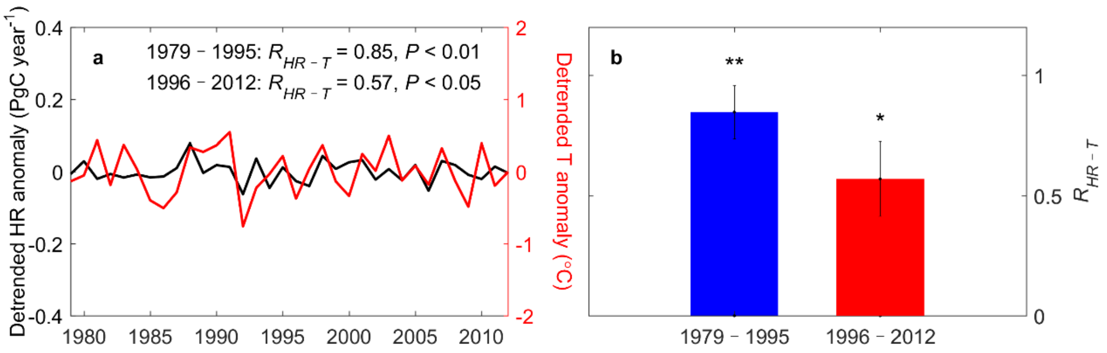

Supplementary Figure 20. (a) The time series of detrended anomaly of summer heterotrophic respiration (HR) derived from Hashimoto et al. (2015) (black line) and summer temperature (T, red line) calculated as the average temperature for July and August across the regions north of 50 °N. (**b**) illustrate the interannual partial correlation coefficient between HR and T. We calculate $R_{HR\text{-}T}$ through randomly selecting 14 of the 17 years in each corresponding period, and then take their standard deviation as the error bar. * and ** indicates that partial correlation coefficient is statistically significant at $P < 0.05$ and $P < 0.01$, respectively.

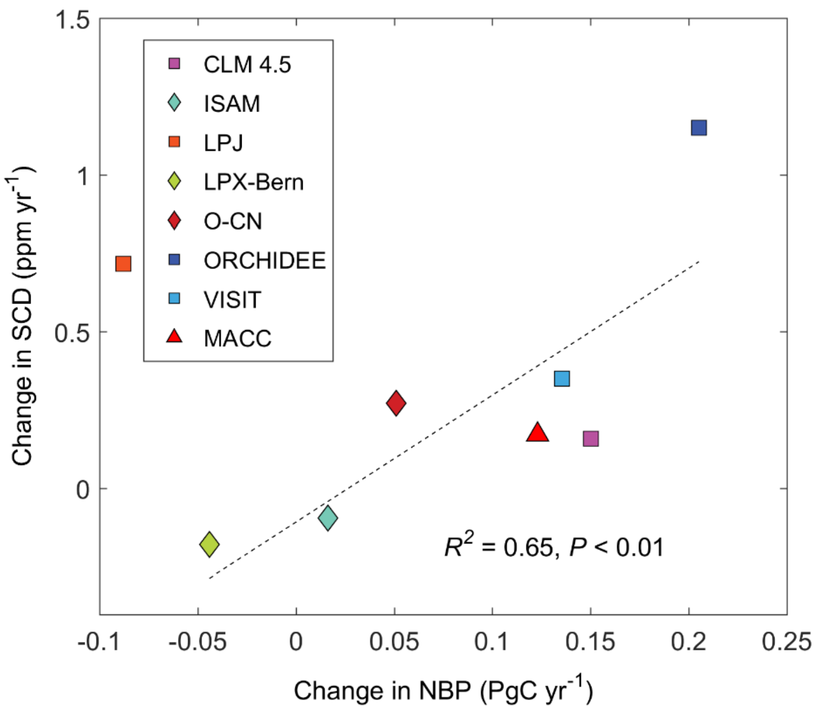

Supplementary Figure 21. The relationship between SCD change and terrestrial net biome productivity (NBP) change. We use LMDZ atmospheric transport model to convert each NBP map into an estimate of atmospheric CO_2 concentration at Barrow station. For each NBP, the corresponding SCD is calculated from the simulated atmospheric CO_2 concentration using LMDZ. The changes in NBP and SCD are computed as the difference between the period 1996–2012 and 1979–1995. Note that the model LPJ was recognized as an outlier and is then not included in the correlation analysis.

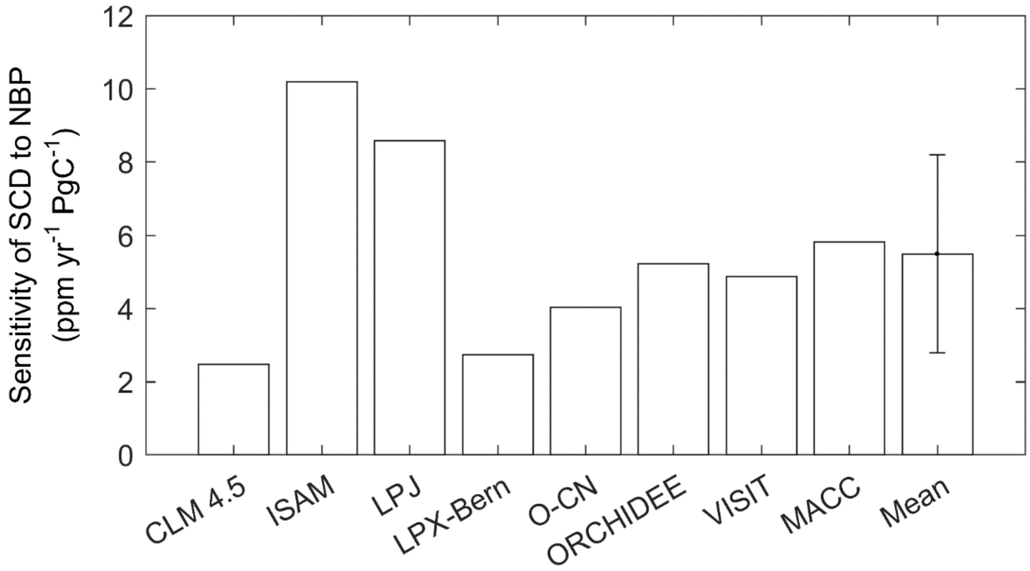

Supplementary Figure 22. The model estimate of sensitivity of SCD change to NBP change based on perturbations of surface fluxes. For each NBP map, we performed control and perturbed simulations. In the perturbed simulation, we increased summer surface fluxes by a scaling value so that summer NBP north of 50°N increased by one unit of PgC for each year of the period 1979–2012. We use LMDZ atmospheric transport model to convert each NBP map into an estimate of atmospheric CO_2 concentration at Barrow station. For each NBP map, the SCDs from control and perturbed simulations are obtained, respectively. The sensitivity is then computed as their difference relative to one unit increase of PgC in terrestrial NBP north of 50°N. The bar and errorbar is the average and standard deviation of sensitivity of SCD to NBP among models.

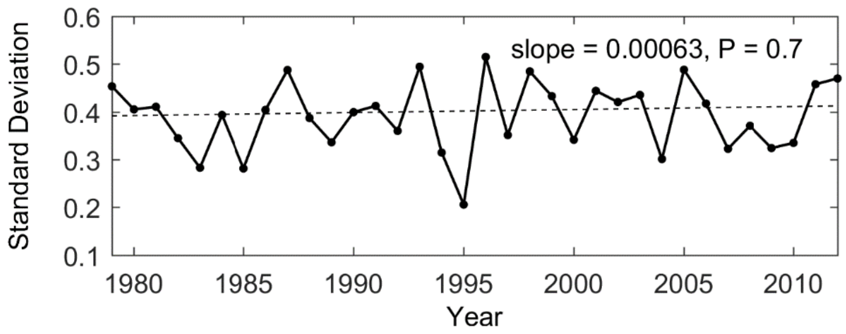

Supplementary Figure 23. The time series of the standard variation of daily temperature between July and August during the period 1979–2012.
